# Tumor-suppressive functions of protein lysine methyltransferases

**DOI:** 10.1038/s12276-023-01117-7

**Published:** 2023-12-01

**Authors:** Nur Aziz, Yo Han Hong, Han Gyung Kim, Ji Hye Kim, Jae Youl Cho

**Affiliations:** Department of Integrative Biotechnology, Sungkyunkwan University, Suwon, 16419 Republic of Korea

**Keywords:** Oncogenes, Cancer genomics

## Abstract

Protein lysine methyltransferases (PKMTs) play crucial roles in histone and nonhistone modifications, and their dysregulation has been linked to the development and progression of cancer. While the majority of studies have focused on the oncogenic functions of PKMTs, extensive evidence has indicated that these enzymes also play roles in tumor suppression by regulating the stability of p53 and β-catenin, promoting α-tubulin-mediated genomic stability, and regulating the transcription of oncogenes and tumor suppressors. Despite their contradictory roles in tumorigenesis, many PKMTs have been identified as potential therapeutic targets for cancer treatment. However, PKMT inhibitors may have unintended negative effects depending on the specific cancer type and target enzyme. Therefore, this review aims to comprehensively summarize the tumor-suppressive effects of PKMTs and to provide new insights into the development of anticancer drugs targeting PKMTs.

## PKMT inhibitors: a double-edged sword in cancer treatment

The review summarizes the tumor-suppressive role of protein lysine methyltransferases (PKMTs), a group of enzymes that add a methyl group to the lysine residues of proteins. The authors highlight three PKMTs – SETD2, SETD7, and EZH2 – which have been found to suppress tumor growth in various types of cancer. For instance, SETD2 has been shown to maintain genomic stability and enhance DNA damage repair in clear cell renal cell carcinoma. Similarly, SETD7 has been found to inhibit cell proliferation and migration in gastric cancer. EZH2, on the other hand, has been shown to suppress tumor growth in lung adenocarcinoma and myelodysplastic syndrome. The authors also discuss the potential of PKMT inhibitors as therapeutic agents for cancer treatment. However, they caution that due to the pleiotropic roles of these enzymes in tumorigenesis, careful consideration is needed when using PKMT inhibitors for cancer therapy.

This summary was initially drafted using artificial intelligence, then revised and fact-checked by the author.

## Introduction

Protein methylation refers to the addition of a methyl group to the ε-amino group of proteins, with S-adenosyl-L-methionine (AdoMet) primarily used as the methyl group donor. Protein residues can be methylated on nitrogen (N-methylation), oxygen (O-methylation), sulfur (S-methylation), and carbon (C-methylation) atoms^1–4^. Lysine methylation is a form of N-methylation that affects histones, playing a central role in histone–protein interactions^5^. The lysine residues of histones H3 and H4 are usually methylated, as represented by histone H3 at lysine 4 (H3K4), lysine 9 (H3K9), lysine 27 (H3K27), lysine 36 (H3K36), and lysine 79 (H3K79) and histone H4 at lysine 20 (H4K20)^6–9^. These residues can be mono- (Kme1), di- (Kme2), or tri- (Kme3) methylated, and histone lysine methylation activates or represses transcription depending on the position of the methylation site. For example, H3K4me3 is a representative active gene mark that is abundantly detected at active transcription start sites (TSSs) and is positively correlated with gene expression^10^. In addition, H3K79me3 is found in transcribed regions of active genes and is reported to correlate with the promotion of gene activation^11^. Conversely, H3K9me3 and H4K20me3 are prominently found in constitutive heterochromatin regions, while H3K27me3 exhibits enrichment in facultative heterochromatin. These epigenetic modifications serve as gene-repressive marks, contributing to the process of gene silencing^12,13^. Gene activation and repression status are also considerably influenced by the extent of methylation. H3K27me1 is an example of a type of methylation with such effects. Unlike H3K27me3, H3K27me1 is detected in both active and repressed chromatin domains, which implies its versatile role in controlling gene expression^14,15^. An additional illustration involves H4K20me1, which is notably abundant within actively transcribing gene bodies. This modification enhances the expression of housekeeping genes by promoting chromatin accessibility, and these effects distinctly contrast with those attributed to H4K20me3^16^.

Recently, due to advances in technologies used to identify methylated proteins, such as mass spectrometry, the range of protein methylation is now known to extend beyond histones to nonhistone proteins^17,18^. According to the PhosphoSitePlus database, 1005 lysine methylation sites in 974 human proteins had been identified as of 2015^19^. The majority of lysine-methylated proteins are transcription factors, such as RelA, STAT, and FOXO3. Kinases or proteins related to phosphorylation also comprise a large proportion of lysine-methylated proteins.

Lysine methylation is a protein lysine methyltransferase (PKMT)-dependent reaction, and lysine residues can be mono-, di-, or trimethylated depending on the type of PKMT. PKMTs play oncogenic roles in various cancers, and preclinical and clinical trials of several PKMT inhibitors for cancer treatment are ongoing^20,21^. However, growing evidence suggests that some methyltransferases exhibit tumor-suppressive functions in specific cancer types and have both oncogenic and tumor-suppressive functions in other cases. Therefore, obtaining a clear understanding of these functions is critical, particularly for developing cancer therapies that target these enzymes. In this review, we discuss the current understanding of PKMTs as tumor suppressors.

## Characteristics of lysine methylation and PKMTs

### Lysine methylation

**Fig. 1:**
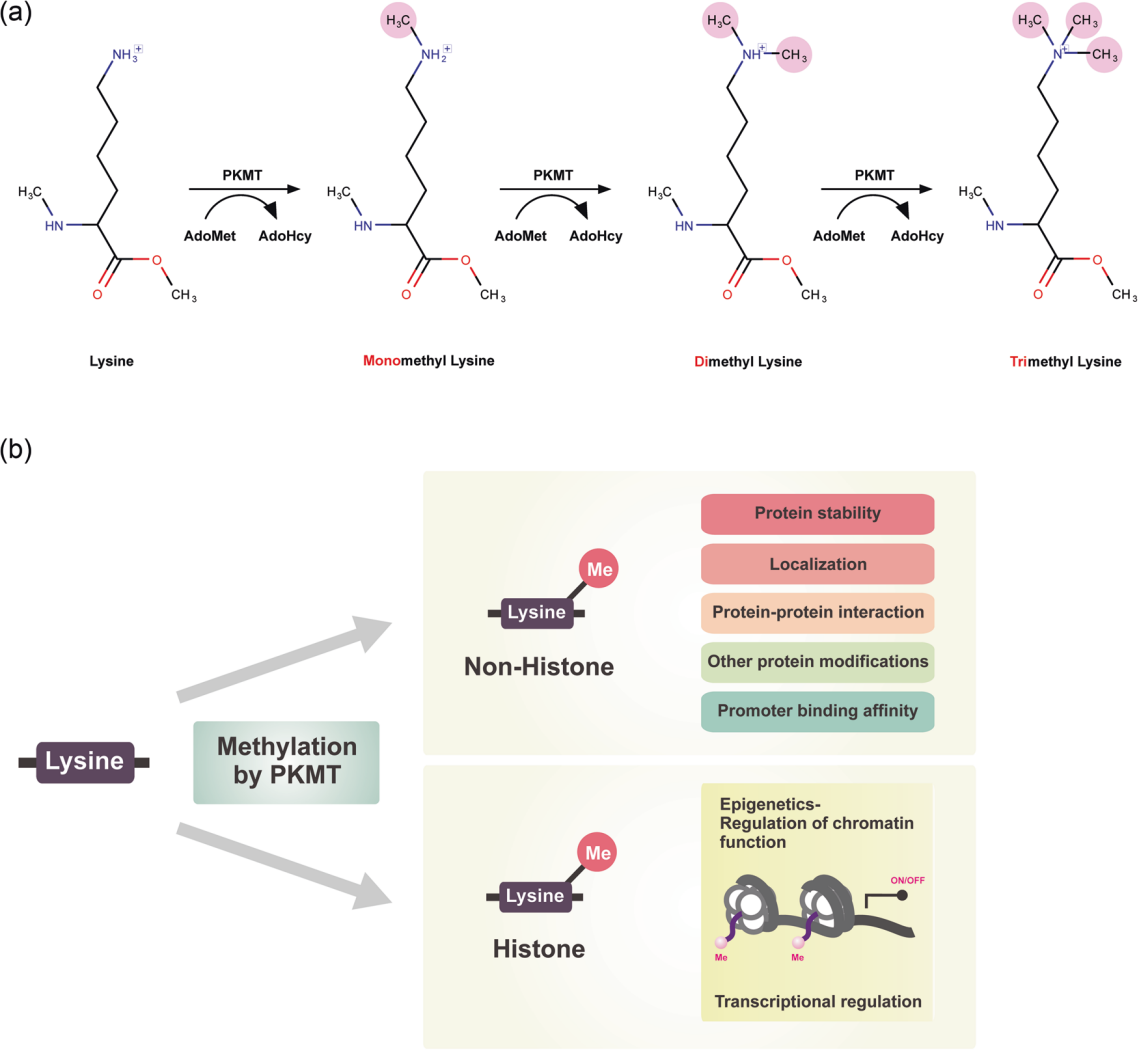
Characteristics of protein lysine methylation. **a** The process of protein lysine methylation. **b** Molecular functions of protein lysine methylation.

Chemically, lysine methylation can be catalyzed by AdoMet-dependent lysine (K)-specific methyltransferases, resulting in the transfer of methyl groups from AdoMet onto lysine residues of particular substrates. Subsequently, AdoMet is converted to S-adenosyl-L-homocysteine (AdoHcy), the demethylated form of AdoMet. The lysine ε-amino acid groups can receive up to three methyl groups, which results in mono-, di-, or trimethylated lysine (Fig. 1a). These distinct methylation states can have different physiological consequences. Lysine methylation can occur on both histone and nonhistone proteins. Histone methylation is associated with changes in chromatin structure and is closely linked to transcriptional regulation. Methylation of nonhistone proteins affects protein stability, cellular localization, protein‒protein interactions, promoter binding affinity, and the regulation of other posttranslational modifications on the substrates (Fig. 1b).

### Classification of PKMTs

Protein methyltransferases are classified into five classes, I, II, III, IV, and V, according to structural similarities of the catalytic domain^22^. PKMTs predominantly belong to the class V superfamily, containing a conserved Su(var)3-9, enhancer of zeste, and trithorax (SET) domain. Methyltransferases containing SET domains are distinguished from other AdoMet-dependent methyltransferases in terms of the available binding sites for substrates and AdoMet^23^. In general, once the substrate binds to the enzyme, the binding of an additional cofactor (e.g., AdoMet) is sterically hindered. However, in methyltransferases with the SET domain, the substrate and AdoMet bind to separate clefts located opposite each other, allowing multiple methylation without dissociation of the substrate^24^. For this reason, it is thought that PKMTs with the SET domain are suitable for trimethylation. As an exception, histone H3 lysine 79 (H3K79) methyltransferase has a disruptor of telomeric silencing 1-like (DOT1L) domain and is classified in a new category called DOT1L domain-containing lysine methyltransferases^25^. DOT1L contains a seven β-strand Rossmann-fold domain, which is commonly observed in class I methyltransferases, such as protein arginine methyltransferases (PRMTs), and is phylogenetically closer to PRMTs than PKMTs^26^.

### Oncogenic role of PKMTs

Numerous PKMTs have been reported to promote tumorigenesis. In particular, SETD7, EZH2, G9a, SMYD2 and SUV4-20H2 have been extensively studied in cancer, and these proteins are being pursued as therapeutic targets of anticancer drugs. Their roles in promoting cancers are well summarized in previous literature^27^. Therefore, we will briefly review their oncogenic functions here.

SETD7 (also known as SET7, SET9, or SET7/9) is highly expressed in hepatocellular carcinoma and colorectal cancer tissues compared to noncancerous tissues, and the increased expression of SETD7 was found to be closely correlated with cancer progression via regulation of cell proliferation and metastasis in these cancers^28,29^. SETD7 is known to stabilize E2F transcription factor 1 (E2F1) in hepatocellular carcinoma, leading to the expression of cyclin E1, cyclin A2, and CDK1, which triggers the cell cycle in tumorigenesis^30^. The carcinogenic role of SETD7 was also identified in breast, intestinal, prostate, and ovarian cancers. In breast cancer, SETD7 facilitates the transcription of vascular endothelial growth factor (VEGF), a key regulator of angiogenesis, by associating with GATA1^31^. In contrast, SETD7-dependent Yes-associated protein (YAP) methylation promotes Wnt-mediated β-catenin activation in intestinal tumorigenesis^32^. Retinoid acid-related orphan receptor α2 (RORα2) is a target of SETD7 in prostate cancer cells^33,34^. In detail, RORα2 methylation at K87 by SETD7 triggers its transcriptional activities by promoting its interaction with the Tip60 coactivator^33^. Furthermore, SETD7 selectively protects hypoxia inducible factor-1α (HIF-1α) from proteasomal degradation only under hypoxic conditions and promotes HIF-1α-mediated gene transcription, which is involved in hypoxic glycolysis. As a result, SETD7 facilitates glycolytic adaptation and the survival of cancer cells under hypoxic conditions. In this process, SETD7 selectively increases H3K4me1 levels in the hypoxia response elements (HREs) of glycolytic genes, thereby promoting the formation of transcriptionally active chromatin^35^. The detailed mechanisms underlying SETD7-mediated HIF-1α stabilization are not yet fully understood.

EZH2 expression is amplified in lymphoma, lung cancer, prostate cancer, breast cancer, colon cancer, melanoma, retinoblastoma, and glioblastoma^36–40^, and EZH2 is involved in the metastasis of prostate and breast cancers^41,42^. EZH2 overexpression can drive tumorigenesis through its role as an epigenetic regulator. Several mechanisms have been proposed to explain the oncogenic role of EZH2, including the repression of tumor suppressor genes such as E-cadherin^43^, CDKN2A/p16^44^, CDKN1A/p21, and CDKN1B/p27^45^. EZH2 can also suppress the activation of oncogenic pathways such as the Wnt/β-catenin pathway by epigenetically repressing GSK-3β and TP53^46^. In addition to its role in tumor initiation and progression, EZH2 has also been implicated in drug resistance. EZH2 can promote drug resistance by enhancing the DNA damage response pathway^47^. Recently, reduction of EZH2 by miR-138 has been proposed as a strategy to overcome drug resistance in multiple myeloma^48^.

The pathological function of EZH2 appears to be context-dependent and cancer-specific^49^. EZH2 cooperates with other oncogenes to accelerate myelodysplastic syndrome, and long-term suppression of EZH2 in glioblastoma causes a significant alteration in cell fate, ultimately leading to tumor progression^50^. However, the tumor-suppressive effect of EZH2 was also observed in diffuse midline glioma^51^. Thus, the features of each tumor should be considered in the application of EZH2 inhibitors. For a more comprehensive understanding of the tumor-suppressive function of EZH2, we kindly direct readers to Section “EZH2 (KMT6)”.

G9a, another oncogenic enzyme that methylates p53^52^, is overexpressed in various cancers, including esophageal squamous cell carcinoma, lung cancer, and aggressive ovarian cancer^53–55^. In addition to p53, elevated G9a under hypoxia has been reported to induce transcriptional repression of runt-related transcription factor 3 (RUNX3), known as a tumor suppressor gene in gastric cancer, via H3K9 dimethylation^56^. Thus, upregulation of G9a ultimately contributes to the aggressive phenotype of cancer cells.

SMYD2, which is highly expressed in esophageal squamous cell carcinoma and bladder cancer cells, is considered an oncogenic protein^57,58^. SMYD2 exhibits an oncogenic effect by repressing the activity of important tumor suppressors, such as p53 and Rb, by methylation of these proteins^59,60^.

SUV4-20H2 favors the mesenchymal state in pancreatic cancer by silencing genes associated with epithelial traits. SUV4-20H2 knockdown induced a transition from a mesenchymal to an epithelial state, reducing stemness and enhancing drug sensitivity. Analysis of pancreatic cancer biopsies confirmed that high SUV4-20H2 levels correlate with loss of epithelial characteristics in invasive cancer. SUV4-20H2 acts as an upstream epigenetic regulator of epithelial/mesenchymal state control, suggesting its potential as a therapeutic target for promoting epithelial identity in cancer^61^.

## Tumor-suppressive role of PKMTs

**Fig. 2:**
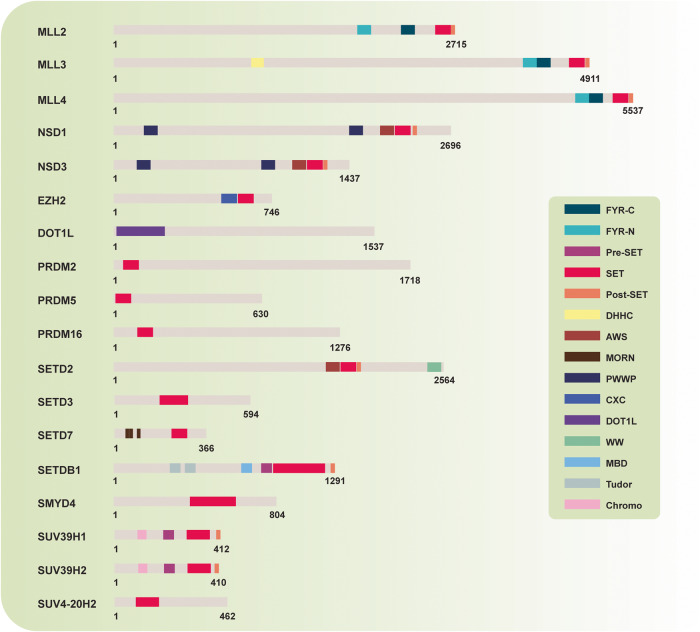
Domain structure of tumor-suppressive protein lysine methyltransferases. This illustration portrays the configuration of domains within lysine methyltransferase proteins, acknowledged for its role in suppressing tumors. Each domain is assigned a distinct color to indicate commonalities or differences among various methyltransferases. The universal presence of the red SET domain in all tumor-suppressive lysine methyltransferases, excluding DOT1L, underscores the pivotal role of their catalytic activity in tumor suppression. Fyr-C Phe/Tyr-rich domain C-terminal, Fyr-N Phe/Tyr-rich domain N-terminal, SET Su(var)3-9 Enhancer-of-zeste Trithorax-domain, DHHC Asp-His-His-Cys containing domain, AWS associated with SET domains, MORN membrane occupation and recognition nexus, PWWP Pro-Trp-Trp-Pro containing domain, CXC Cys-rich domain, DOT1L catalytic domain of DOT1L, WW domain with 2 conserved Trp-Trp residues, MBD methyl-CpG binding domain, Tudor tudor domain, Chormo Chromatin organization modifier domain.

**Fig. 3:**
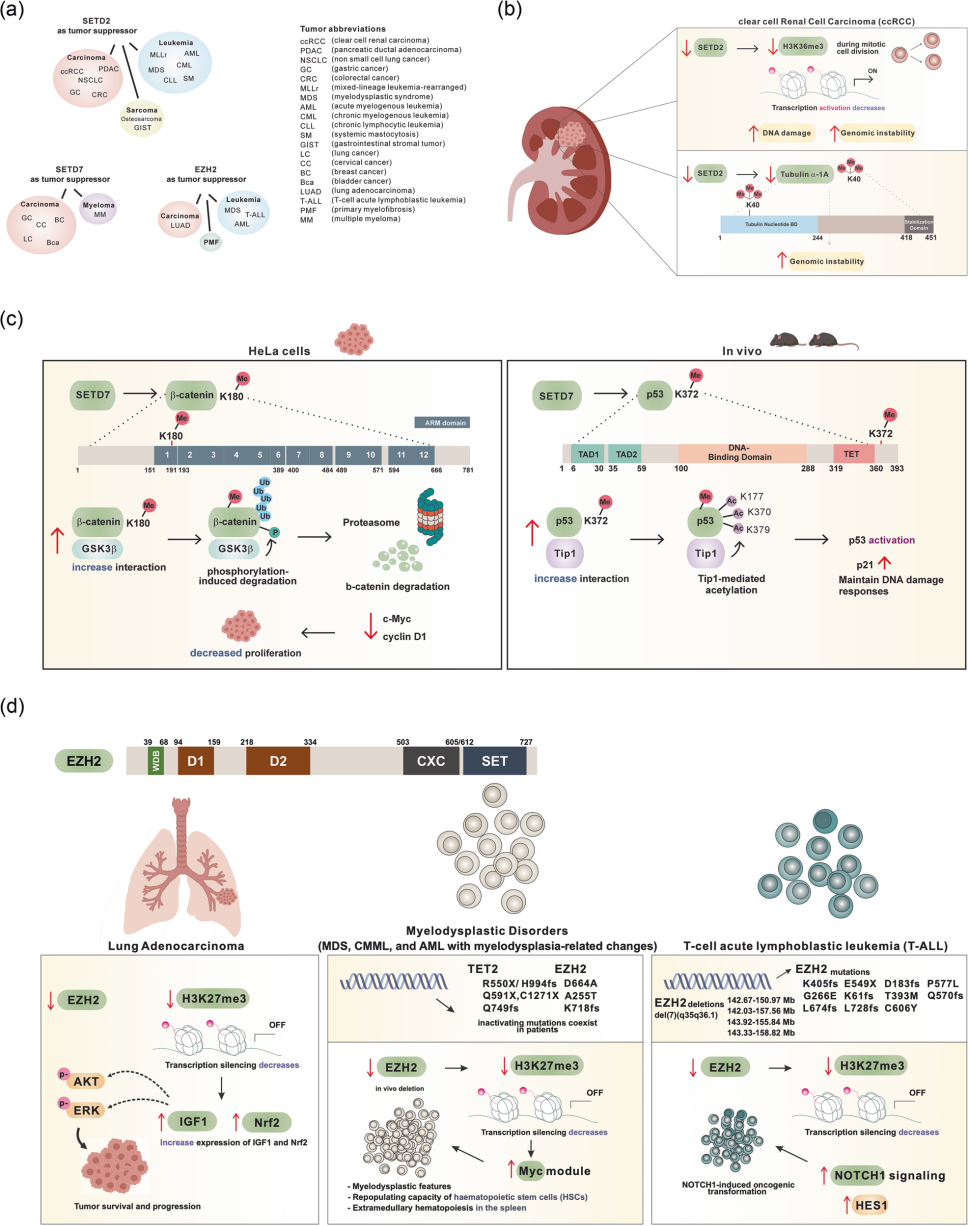
The context-dependent tumor-suppressive effect of methyltransferases. **a** Various tumor types controlled by three lysine methyltransferase variants that are frequently linked to tumor suppressor functions. **b** Mechanistic role of SETD2 as a tumor suppressor in clear-cell renal cell carcinoma. Reduction in SETD2 levels results in a decrease in H3K36 trimethylation and tubulin α-1a trimethylation, which subsequently initiates genomic instability. **c** The mechanism of action of SETD7 as a tumor suppressor. Left: SETD7 catalyzes the monomethylation of β-catenin at K180, amplifying its molecular interaction with GSK3β. This prompts GSK3β-mediated phosphorylation, leading to ubiquitination of β-catenin followed by proteasomal degradation in HeLa cells. Consequently, this process leads to diminished levels of c-Myc and cyclin D1, resulting in a decrease in cell proliferation. Right: In an in vivo model, SETD7 initiates monomethylation of p53 at K372, leading to its interaction with Tip1. This interaction subsequently induces acetylation of p53, resulting in the activation of p53 itself. This activation leads to heightened levels of p21 and the preservation of the DNA damage response. **d** Upper: Structure of EZH2 with domain WDB, WD-40 binding domain; D1, domain 1; D2, domain 2; CXC, Cys-rich domain; SET, catalytic domain of EZH2. Lower: The mechanistic role of EZH2 as a tumor suppressor in lung adenocarcinoma, myelodysplastic disorders, and T-cell acute lymphoblastic leukemia (T-ALL) through its involvement in modulating the trimethylation status of H3K27. Decreased expression and mutation of EZH2 (as depicted) have been identified in individuals with lung adenocarcinoma, myelodysplastic syndromes, and T-ALL. The attenuation of EZH2 expression or activity leads to a reduction in H3K27me3 levels, resulting in enhanced activation of downstream proteins such as AKT, ERK, Nrf2, Myc, and NOTCH1, which are implicated in the development of these conditions.

Extensive literature reviews in the field of protein lysine methylation have reported on its cellular functions, including epigenetic regulation, signaling pathways, and various cellular responses. However, literature reviews on the tumor-suppressive functions of PKMTs are lacking. Eighteen PKMTs have been identified as tumor suppressors, and their conserved domains are depicted in Fig. 2. These PKMTs facilitate the methylation of various substrates, such as p53, β-catenin, α-tubulin, and histone proteins. The tumor-suppressive functions of PKMTs are mediated through methylation of these substrates. Among these PKMTs, SETD2, SETD7, and EZH2 are relatively well characterized with dominant tumor-suppressive functions (Fig. 3a). In this article, we highlight the substrates and tumor-suppressive functions of PKMTs in various human cancers (Table 1).

**Table 1:** Molecular characteristics and tumor-suppressive functions of PKMTs.

Enzyme name	Family	Conserved domain and motif (Positions)	Subcellular localization	Histone substrates	Nonhistone substrates	Cancer type	Tumor-suppressive functions	References
SETD2 (SET2, KMT3A)	SET2 subfamily	AWS domain (1494–1548), SET domain (1550–1667), post-SET domain (1674–1690), LCR domain (2137–2366), WW domain (2389–2422), SRI domain (2469–2548)	Nuclear speckles, cytosol	H3K36me3	α-Tubulin K40me3	ccRCC	Trimethylates α-tubulin at lysine 40, maintaining genomic stability.	^70–72^
							Promotes ATM activation upon DSBs and enhances homologous recombination repair of DSBs in an H3K36me3-dependent manner.	^73^
							Depletion upregulates oncogenes in an H3K36me3-dependent manner.	^74^.
							Depletion inhibits the cell metabolic pathway in an H3K36me3-dependent manner.	^74,75^
						MLLr	Loss downregulates tumor suppressors and upregulates oncogenes.	^78^
						MDS	Deficiency enhances hematopoietic stem cell signaling and decreases myeloid differentiation pathways.	^79^
							Downregulates S100a9 in an H3K36me3-dependent manner.	
						CLL	Depletion induces loss of TP53, genomic complexity, and chromothripsis.	^80^
						SM	Recovery by a proteasome inhibitor (bortezomib) induces apoptosis and inhibits colony formation when combined with midostaurin.	^66^
						AML	Mutations confer resistance to DNA-damaging agents.	^82^.
							Deficiency destroys DNA damage response to cytotoxic chemotherapy.	
							Loss-of-function mutations cause resistance to conventional cytarabine-based chemotherapy.	^83^
							Mutations downregulate cell cycle-related signal.	
						CML	Depletion enhances resistance to imatinib by upregulating oncogenes.	^84^
						Pancreatic ductal adenocarcinoma	SETD2 loss promotes KRAS-induced ductal metaplasia via dysregulation of E3 ligase Fbxw7.	^85^
							SETD2 loss induces EMT through dysregulation of catenin α1	
							SETD2 loss promotes metastasis via AKT activation.	
							SETD2 deficiency facilitates immune evasion.	^86^
						Lung cancer	SETD2 depletion upregulates oncogenes.	^88^
							SETD2 is involved in cisplatin-induced apoptosis through regulation of the ERK signal.	^89^
							SETD2 inactivation enhances mTORC1-associated gene expression.	^90^.
						Gastric cancer	SETD2 suppresses cell proliferation, migration, and invasion.	^91^.
						Gastrointestinal stromal tumor	SETD2 inhibits rH2Ax.	^92^.
						Colorectal cancer	SETD2 reduces WNT signal-mediated tissue regeneration and tumorigenesis.	^93^.
						Osteosarcoma	SETD2-mediated H3K36me3 is involved in resistance to cisplatin through upregulation of GSK3β and degradation of β-catenin.	^96^
SETD7 (SET7/9, KMT7)	SET7 subfamily.	MORN 1 ~ 3 domain (36–58, 59–81, 106–128), SET domain (214–336), post-SET domain (345–366)	Nucleoli	H3K4me1	p53 K372me1, β-catenin K180me1	–	Methylates p53 at lysine 372, leading to p53 activation.	^98^.
						Cervical cancer	Methylates β-catenin at lysine 180, resulting in β-catenin instability. In addition, this modification eventually causes downregulation of Wnt/β-catenin target genes such as c-Myc and cyclin D1.	^100^
						Gastric cancer	Reduces cell proliferation, migration, and invasion.	^101^.
							Upregulates gene expression, including SREK11P1, PGC, and CCDC28B, in an H3K4me-dependent manner.	
						Multiple myeloma	Involved in berberine-induced apoptosis by mediating the proteasome-dependent degradation of NF-κB.	^102^.
						Breast cancer	Deficiency enhances stability of E2F1 protein, leading to epithelial mesenchymal and cancer stem cell-like properties.	^103^
							Inhibits tumorigenesis via regulation of Gli-1 expression.	^104^
						Lung cancer	Suppresses metastasis through the JAK2/STAT3 pathway.	^105^.
						Bladder cancer	SETD7 mRNA decay by METTL3 promotes cell proliferation and metastasis.	^106^.
EZH2 (KMT6)	SET3 subfamily, polycomb-repressive complex 2	CXC domain (503–605), SET domain (612–727)	Nucleoplasm	H3K27me, H3K27me3	–	Lung adenocarcinoma	Loss of function increases the development of tumors via activation of AKT and ERK in an IGF1 signaling-dependent manner.	^107^
							Reduces cell proliferation and colony formation through inhibition of Nrf2 expression.	^108^
						MDS	Deficiency, in conjunction with loss of Tet2 or RUNX1, contributes to development of MDS.	^112,113^.
						PMF	Depletion activates Hmga2 genes in concert with the JAK2V617F mutation.	^116^
						T-ALL	Depletion promotes development of T-ALL.	^117^
							Activity is suppressed by NOTCH1, suggesting interplay between EZH2 and NOTCH1.	^118^
						AML	Deficiency induces resistance to tyrosine kinase inhibitors by HOX expressions.	^119^.
MLL3 (KMT2C)	TRX/MLL subfamily	DHHC domain (436–489), FYR domain (4545–4691), SET domain (4771–4887), post-SET domain (4895–4911), WIN motif (4707–4712)	Nucleoplasm	H3K4me1, H3K4me2, H3K4me3	–	AML	Deficiency accelerates the development of myeloid leukemia in p53-suppressive conditions.	^149^
							Downregulation is involved in resistance to conventional chemotherapy.	^150^
						Bladder cancer	Depletion dysregulates the DNA damage response.	^153,154^
							Low MLL3-expressing cells rely on PARP1/2 for repair of DNA. Thus, olaparib, a PARP1/2 inhibitor, is proposed as a therapeutic intervention to induce synthetic lethality in these cells.	
PRDM2/RIZ (KMT8)	Class V like SAM binding methyltransferase superfamily	SET domain (28–141), SH3-binding motif (970–979, 985–998, 1028–1052)	Nucleoplasm, Golgi apparatus	H3K9me2	–	Malignant meningioma, somatotroph adenoma	Induces cell cycle arrest and apoptosis in a c-Myc-dependent manner.	^158,160,163^
						Glioma	Induces apoptosis and cell cycle arrest by upregulating NF-κB and AKT signals.	^161^.
						Breast cancer, liver cancer, colon cancer	Induces cell cycle arrest and apoptosis.	^159,166,167^
						Endometrial cancer	Depletion promotes tumor growth, migration, and invasion.	^169^.
						Testicular germ cell tumors	The RIZ1 gene is located in the chromosomal region frequently deleted in this cancer, suggesting its tumor-suppressive role. However, detailed molecular mechanisms are not understood.	^170^
						CML	In an H3K9me2-dependent manner, it modulates insulin-like growth factor-1 signaling to induce apoptosis and differentiation, while simultaneously repressing cell proliferation.	^172^
PRDM5	Class V-like SAM binding methyltransferase superfamily	SET domain (8–124)	Nucleoplasm, nuclear bodies, nucleoli	H3K27me3	–	Colorectal cancer	Suppression is linked to advanced stages across colorectal polyps.	^173^
						Ovarian cancer, breast cancer, hepatoma	Facilitates cell cycle arrest and apoptosis.	^175^
						Intestinal cancer	Depletion promotes the development of adenomas and microinvasion by dysregulating expression of metabolic pathway-related genes.	^176^
						Multiple cancer	Inhibits tumorigenesis by regulation of the Wnt/β-catenin pathway.	^177^
SMYD4	Class V-like SAM binding methyltransferase superfamily	TPR1 domain (68–171), MYND domain (296–335), SET domain (233–574), TPR2 domain (660–726)	Nucleoplasm, Golgi apparatus, vesicles, cytosol	H3K4me2, H3K4me3	–	Breast cancer	Suppresses tumorigenesis by mediating inhibition of PDGFR-α expression.	^178^
							miR-1307-3p-targeting SMYD4 promotes malignant transformation of mammary epithelial cells.	^180^
						Renal cell carcinoma, glioblastoma	Copy number alteration is observed.	^181,182^,.
SUV4-20H2 (KMT5C)	Suvar4-20 subfamily.	SET domain (102–212), post-SET domain (212–249)	Nucleoplasm	H4K20me1, H4K20me2, H4K20me3	CASZ1 K1423, OIP5 K215, CENPU K308	Breast cancer	Inhibits metastatic ability by decreasing metastasis-related genes, such as tensin-3.	^183^
							miR-29a-mediated suppression accelerates metastasis by upregulation of CTGF and EGR1.	^184^
						Lung cancer	Depletion leads to cancer progression via dysregulation of telomere length maintenance.	^186^.
						Liver cancer, osteosarcoma	Upregulated in these types of cancers.	^185,187^.
EEF1AKMT3 (METTL21B)	Seven-beta-strand methyltransferase family, Family 16 methyltransferase	The domain structure is not elucidated yet.	Nucleus, cytoskeleton, cytosol		EEF1A, MAP2K7	Gastric cancer	Inhibits invasion and metastasis by TP53 stabilization.	^188^

*ALL* acute lymphoblastic leukemia, *AML* acute myeloid leukemia, *ccRCC* clear cell renal cell carcinoma, *CLL* chronic lymphocytic leukemia, *CML* chronic myeloid leukemia, *CRC* colorectal cancer, *EATL* enteropathy-associated T-cell lymphoma, *GIST* gastrointestinal stromal tumor, *LUAD* lung adenocarcinoma, *MCL* mast cell leukemia, *MDS* myelodysplastic syndrome, *NPC* nasopharyngeal cancer, *NSCLC* non-small cell lung cancer, *PDAC* pancreatic ductal adenocarcinoma, *PMF* primary myelofibrosis, *SM* systemic mastocytosis, *T-ALL* T-cell acute lymphoblastic leukemia, *testicular GCT* testicular germ cell tumor, *CTGF* connective tissue growth factor, *EGR1* early growth response 1, *IGF1* insulin-like growth factor 1, *Nrf2* nuclear factor E2-related factor, *PDGFR-α* platelet-derived growth factor α.

### SETD2 (SET2, KMT3A)

SET domain-containing 2 (SETD2) is part of the SET2 subfamily, histone lysine methyltransferase family, and class V-like SAM binding methyltransferase superfamily. SETD2 consists of several domains, including the associated with SET (AWS) domain; the SET domain; the post-SET domain; the locus control region (LCR); the WW domain, which binds to a proline-rich motif of a protein; and the Set2 Rpb1-interacting (SRI) domain, which is crucial for transcriptional elongation through histone H3 lysine 36 (H3K36) methylation (Fig. 2). SETD2 is located on chromosome 3p21 and facilitates the trimethylation of H3K36 and microtubules at lysine 40 (αTubK40). Nuclear receptor-binding SET domain protein 1 (NSD1) primarily catalyzes the demethylation of H3K36, while NSD2 (also known as WHSC1 or MMSET) acts as a mono- and demethylase of H3K36^62–65^. This demethylation provides a substrate for trimethylation by SETD2. In addition, the SETD2 protein level is regulated by miR-106-5p, ubiquitination, and SUMOylation^66,67^. The R1625 residue in the catalytic domain of SETD2 is important for its interactions with the histone H3 tail and the trimethylation of H3K36 but not for its thermal stability^68^. Reader proteins that recognize the H3K36me3 modification recruit protein complexes involved in transcription elongation, RNA processing, DNA repair, and other processes^69^.

In clear cell renal cell carcinoma (ccRCC), SETD2 mediates the trimethylation of α-tubulin at K40, which is required to maintain genomic stability, and loss of SETD2 function results in genomic instability and contributes to ccRCC tumorigenesis. Specifically, SETD2 deficiency and haploinsufficiency led to ccRCC progression through an increase in mitotic defects and micronuclei formation (Fig. 3b)^70,71^. In addition, loss of function of SETD2 facilitates ccRCC progression through dysregulation of genome integrity-related processes, including nucleosome destabilization, replication stress, and DNA repair^72^.

SETD2 suppresses ccRCC development through distinct mechanisms mediated by H3K36 trimethylation. SETD2-mediated H3K36me3 promotes ataxia telangiectasia mutated kinase (ATM) activation on DNA double-strand breaks (DSBs) and enhances homologous recombination repair of DSBs by accelerating the formation of RAD51 presynaptic filaments. In contrast, the loss of SETD2 impaired DNA damage signaling, including inactivation of p53, despite the persistence of DNA lesions^73^. In addition, genetic depletion of SETD2 upregulated oncogenes and inhibited cell metabolic pathways^74^. This change also enhanced overall metabolic activity, including ATP and glycolytic activity, mitochondrial respiratory capacity, TCA cycle metabolic enzyme activity, and production of TCA metabolites, through PGC1α, a central regulator of mitochondrial oxidative phosphorylation and fatty acid metabolism^75^.

In addition, SETD2 knockdown prevented cyclin-dependent kinase inhibitor 2A (CDKN2A)-induced cellular senescence of renal primary tubular epithelial cells (PTECs) through activation of E2F signaling pathways^76^. Finally, inhibition of miR-106-5p-mediated SETD2 downregulation promoted cell cycle arrest at the G0/G1 phases and caspase-3-mediated apoptosis in ccRCC^67^. Hematopoietic stem cells in *Setd2* knockout mice exhibited impaired self-renewal but overcame the growth disadvantage after a latency period and eventually showed characteristics of hematopoietic malignancy. Mechanistically, the loss of SETD2 in hematopoietic stem/progenitor cells triggers the activation of the Klf1-related pathway; Klf1 is an erythroid transcription factor responsible for the development of hematopoietic malignancies. In addition, SETD2 deficiency activates the E2F gene regulatory network and suppresses the expression of the ribonucleotide reductase subunit Rrm2b, leading to DNA replication stress in hematopoietic stem cells, cellular abnormalities, and genomic instability^77^. These biological functions of SETD2 in hematopoietic stem cells provide insight into the role of SETD2 as a tumor suppressor.

The tumor-suppressive function of SETD2 is observed in various hematopoietic malignancies. SETD2 loss-of-function mutations were found in 22% of patients with leukemia with rearrangements in the mixed-lineage leukemia gene (referred to as MLLr); altered histone methylation due to SETD2 knockdown transcriptionally downregulated tumor suppressors (ASXL1, ASXL2, BCOR) and upregulated oncogenes (ERG, STAT3, FNDC3A, NAMPT, IGF1R)^78^. In addition, SETD2 deficiency enhanced hematopoietic stem cell signaling and decreased myeloid differentiation pathways in hematopoietic stem and progenitor cells from NUP98-hoxd13 (NDH13) transgenic mice in a myelodysplastic syndrome (MDS) model^79^. In that study, downregulation of S100a9 by SETD2 contributed to MDS pathology in an H3K36me3-dependent manner^79^. In chronic lymphocytic leukemia (CLL), SETD2 deletions were related to loss of TP53, genomic complexity, and chromothripsis^80^. Finally, decreased or absent SETD2 protein expression and reduced levels of H3K36 trimethylation have been observed in systemic mastocytosis (SM), with low expression of SETD2 correlating with disease aggressiveness. Notably, the use of the proteasome inhibitor bortezomib is effective in restoring SETD2 expression and H3K36 trimethylation. Furthermore, restoration of SETD2 expression via bortezomib resulted in the induction of apoptosis and inhibition of colony formation when combined with midostaurin, the primary therapeutic option for advanced SM^66^.

Loss of SETD2 is associated with chemoresistance in MLLr leukemia, acute lymphocytic leukemia (ALL), and chronic myelogenous leukemia (CML). The incidence of mutations in SETD2 is higher in cases of relapsed ALL and MLLr acute leukemia^81,82^. Mutations in SETD2 confer resistance to DNA-damaging agents, such as cytarabine, 6-thioguanine, doxorubicin, and etoposide, while not affecting the response to non-DNA-damaging agents^82^. Additionally, loss of SETD2 leads to decreased activation of the DNA damage response (DDR) to cytotoxic chemotherapy, ultimately resulting in reduced apoptosis^82^. Consistently, SETD2 loss-of-function mutations cause resistance to conventional cytarabine-based chemotherapy. Specifically, leukemic cells carrying SETD2 mutations exhibit inhibition of signaling pathways involved in cell cycle progression and regulation of the S and G2/M checkpoints^83^. Consequently, these cells exit the S phase and progress to the G2/M phase after cytarabine treatment^83^. In addition, SETD2 deficiency enhances resistance against imatinib in CML cells by upregulating novel oncogenes, such as erythroblast transformation‐specific (ETS)‐related gene (ERG) and N‐myc proto-oncogene protein (MYCN)^84^. In addition, restoration of H3K36me3 expression by the demethylase inhibitor JIB-04 resensitizes CML to imatinib^84^. Taken together, the findings from these studies indicate that recovery of SETD2 may be a potential therapeutic strategy for overcoming chemotherapy-resistant hematopoietic malignancies.

Deficiency of SETD2 has been implicated in tumorigenesis in several other cancers. In pancreatic ductal adenocarcinoma (PDAC), SETD2 ablation facilitated inflammation and Kras-induced ductal metaplasia by impairing epigenetic regulation of the E3 ligase Fbxw7^85^. In addition, SETD2 loss induced epithelial-mesenchymal transition (EMT) via epigenetic dysregulation of catenin α1^85^. Moreover, SETD2 depletion promoted metastasis through sustained protein kinase B (AKT) activation^85^. In addition, SETD2 deficiency induced neutrophil reprogramming toward an immunosuppressive phenotype, which facilitated immune evasion of pancreatic cancer progression^86^.

SETD2 loss promoted progression in early- and late-stage lung adenocarcinoma in a KRAS^G12D^-driven mouse model^87^. A recent report showed that knockout of SETD2 increases the transcriptional regulation of oncogenes by influencing chromatin accessibility and histone chaperone recruitment, leading to accelerated KRAS^G12D^-driven lung tumorigenesis^88^.

In non-small cell lung cancer (NSCLC), SETD2-mediated H3K36me3 is essential for cytotoxicity to cisplatin through regulation of the extracellular signal-regulated kinase (ERK) signaling pathways^89^. Moreover, SETD2 inactivation enhanced mTORC1-associated gene expression in a KRAS-driven lung adenocarcinoma model in vivo, whereas mTORC1 inhibition enhanced therapeutic susceptibilities in SETD2-inactivated cancer^90^. These findings collectively suggest that mutations in SETD2 confer therapeutic resistance to lung cancer.

SETD2 significantly inhibited cell proliferation, migration, and invasion in gastric cancer^91^. In gastrointestinal stromal tumors (GISTs), SETD2 inhibited rH2Ax, a DNA damage marker, and its mutations have been linked to upregulation of HOXC cluster genes and hypomethylated heterochromatin^92^. Additionally, in colorectal cancer (CRC), SETD2 suppressed WNT signal-mediated tissue regeneration and tumorigenesis, including colony formation, cell proliferation, migration, self-renewal, and stemness, through epigenetic regulation of RNA processing^93^. Furthermore, the majority of SETD2 mutations coincide with mutations in TP53 or KRAS^94^, which suggests that SETD2 mutations disrupt DNA repair mechanisms by destabilizing TP53, augmenting their oncogenic effects in CRC^94^.

In an in vivo 7,12-dimethylbenz[a]anthracene (DMBA)-induced breast cancer model, SETD2 gene and protein expression levels were significantly decreased in very early-stage (13 weeks) and full-fledged tumors (6 months)^95^. In addition, in osteosarcoma, SETD2 significantly inhibited cell growth, cancer stem cell properties, and cisplatin-induced chemoresistance through H3K36me3-mediated transcriptional activation of GSK3β and degradation of β-catenin^96^.

SETD2 is currently considered a potential tumor suppressor. However, a recent study revealed that artificially inducing excessive accumulation of SETD2 in HEK293T cells triggers the activation of signals associated with the cell cycle, leading to an oncogenic phenotype characterized by increased cell proliferation and migration^97^. This finding emphasizes the importance of comprehending the regulatory mechanisms concerning SETD2 that govern tumor development and progression in various types of carcinomas. This knowledge is essential to effectively utilize SETD2 as a therapeutic biomarker while mitigating the risk of unforeseen adverse effects. Moreover, this finding indicates that the cellular and molecular environments of a cell, a tissue, and an organ can affect whether a gene is functionally oncogenic or tumor suppressive. Thus, more studies on SETD2 and protein lysine methyltransferase genes are required for their application as therapeutic targets.

### SETD7 (SET7/9, KMT7)

SETD7, a histone lysine methyltransferase, is included in the SET7 subfamily, histone lysine methyltransferase family, and class V-like SAM binding methyltransferase superfamily. The SETD7 gene is located on chromosome 4q31.1 and encodes the SETD7 protein, which contains 366 amino acids including MORN1/2/3, SET, and post-SET domains (Fig. 2). This enzyme has been reported to have both histone and nonhistone substrates, including H3K4, p53, and β-catenin.

Multiple reports have indicated that SETD7 exhibits tumor-suppressive functions. In SETD7 knockout mice, SETD7 was found to facilitate the monomethylation of p53 at lysine 372 (Fig. 3b)^98^. This methylation is critical for activating p53 and enabling its acetylation by Tip60. In addition, defects in SETD7 led to an impaired p53 response to DNA damage^98^. However, mice lacking SETD7 failed to develop tumors within 1 year, while 74%-83% of p53-deficient mice developed tumors within 6 months^99^. This observation may be because the SETD7 mutation in mice does not result in complete inactivation of the p53 protein or because SETD7 induces the activation of an alternative compensatory pathway.

In cervical cancer, monomethylation of β-catenin at lysine 180 (β-catenin K180me1) is mediated by SETD7 (Fig. 3c). This modification reduces the stability of β-catenin through ubiquitination induced by GSK3β, which leads to a decrease in cancer cell proliferation. This effect eventually leads to downregulation of Wnt/β-catenin target genes, such as c-Myc and cyclin D1^100^. In gastric cancer, a reduced expression level of SETD7 was observed in 34.3% (129/376) of patients and was significantly correlated with clinical aggressiveness and a poor prognosis (*p* < 0.05). SETD7 enhances the gene expression of SREK1IP1, PGC, and CCDC28B through monomethylation of H3K4, significantly decreasing cell proliferation, migration, and invasion^101^. In multiple myeloma, berberine, a naturally occurring isoquinoline alkaloid, exhibited anticancer activity by mediating the upregulation of SETD7. Elevated SETD7 resulted in proteasome-dependent degradation of NF-κB and promoted berberine-induced apoptosis^102^. In breast cancer, a negative regulatory loop involving SETD7 and DNA methyltransferase 1 (DNMT1) has been observed. Specifically, SETD7 was found to negatively regulate the stability of the DNMT1 protein, leading to repression of the transcriptional activity of the SETD7 promoter through coordination with Snail. Additionally, SETD7 deficiency enhanced the stability of the E2F1 protein, promoting epithelial mesenchymal and cancer stem cell-like properties^103^. Furthermore, SETD7 suppressed the proliferation, migration, and invasion of breast cancer cells through regulation of Gli-1 expression^104^. These findings suggest that loss of SETD7 is an early prognostic marker for metastasis in breast cancer.

SETD7 also exhibits tumor-suppressive effects in lung and bladder cancer. In lung cancer, SETD7 suppresses migration and invasion by downregulating the JAK2/STAT3 pathway^105^. In bladder cancer, the m6A modification of SETD7 mRNA by METTL3 leads to mRNA decay, which facilitates cell proliferation and metastasis^106^.

### EZH2 (KMT6)

Enhancer of zeste homolog 2 (EZH2) is a subunit of polycomb-repressive complex 2 (PRC2) and belongs to the polycomb complex, SET3 subfamily, histone lysine methyltransferase family, and class V-like SAM binding methyltransferase superfamily. The EZH2 gene is situated on chromosome 7q36 and encodes a protein with CXC and SET domains (Fig. 2). EZH2 catalyzes di- and trimethylation of histone H3 at lysine 27 (H3K27), as well as the monomethylation of GATA binding protein 4 (GATA4) at lysine 299 (GATA4 K299).

In lung adenocarcinoma, loss of function of EZH2 was observed to increase the number of tumor lesions, which was attributed to activation of AKT and ERK through insulin-like growth factor 1 (IGF1) signaling. Furthermore, loss of EZH2 accelerated inflammatory responses, as evidenced by increased infiltration of macrophages and neutrophils, along with secretion of tumor-associated cytokines, such as interleukin (IL)-6 and tumor necrosis factor-alpha (TNF-α)^107^. Additionally, EZH2 was shown to suppress cell proliferation and colony formation in NSCLC by inhibiting the expression of nuclear factor E2-related factor 2 (Nrf2)^108^.

Homozygous EZH2 mutations (9/12) are associated with 7q acquired uniparental disomy (aUPD) in myeloid malignancies. The mutations are predominantly present in individuals with MDS/MPNs (12% of 219 individuals) or primary myelofibrosis (PMF) (13% of 30 individuals). These mutations are believed to contribute to the development of myeloid malignancies by causing premature chain termination and direct abrogation of methyltransferase activity^109^. Loss-of-function mutations in EZH2 are associated with poor prognosis in patients with MDS and MPN^110,111^. Consistent with clinical outcomes, studies of mice have demonstrated that loss of EZH2 in combination with loss of Tet2 or RUNX1 can promote the development of MDS^112,113^. Approximately 10% of MPN patients with PMF have EZH2 loss-of-function mutations, while JAK2^V617F^, a JAK-activating mutation, was identified in 50% of PMF patients with EZH2 mutations^111,114^. Previous reports have shown that acquisition of the JAK2^V617F^ mutation alone is insufficient for malignancy initiation in vivo^115^. However, loss of EZH2 in JAK2^V617F^ hematopoietic cells reduced the level of H3K27me3 and resulted in conversion of H3K27 to acetylation. This epigenetic alteration activates Hmga2, a signature gene implicated in PMF pathogenesis^116^. It is thus hypothesized that EZH2 dysfunction and JAK2 activation together play an essential role in the pathogenesis of PMF.

In addition to its established role in myeloid malignancies, EZH2 has been implicated in the development of T-cell acute lymphoblastic leukemia (T-ALL) and acute myeloid leukemia (AML). EZH2 deletion has been shown to promote the spontaneous development of T-ALL and decrease survival rates^117^. In T-ALL, EZH2-mediated H3K27me3 was disrupted by NOTCH1 activation^118^. In contrast, CDK1-mediated phosphorylation of EZH2 at Thr487 led to proteasome-dependent degradation of EZH2, the stability of which is due to HSP90 in AML. Moreover, downregulation of EZH2 induced resistance to tyrosine kinase inhibitors via HOX gene expression^119^.

In addition to its tumor-suppressive roles, EZH2 has been extensively implicated in the development of cancer. High expression of EZH2 was found to be a feature of several solid cancers, such as prostate cancer^37,41^, melanoma^37^, endometrial carcinoma^37,120^, breast cancer^37^, esophageal cancer^121^, gastric cancer^122^, anaplastic thyroid carcinoma^123^, and nasopharyngeal carcinoma^124^. Moreover, the interplay between the expression of EZH2 and BAP-1 (BRCA1-associated protein 1) holds significant implications for epigenetic regulation and regulation of cellular processes. BAP-1 is a deubiquitinating enzyme that participates in histone modification removal, DNA repair, and other essential cellular functions^125^. Although a direct link between BAP-1 and EZH2 has not been identified, clinical studies have shown that enhanced EZH2 expression is observed in renal clear cell carcinoma with lower BAP-1 expression and poor prognosis^126^, and loss of BAP1 function increases the efficacy of EZH2 inhibitors^127,128^. Elevated expression of EZH2 was also detected in rhabdomyosarcoma^129^ and Ewing sarcoma^130^ tissues. In the case of lymphomas, the expression level is not significantly altered, likely due to high basal expression in normal proliferating B cells^131^.

Mutations specifically affecting the tyrosine 641 (Y641) residue within EZH2’s catalytic SET domain have a notable impact on its enzymatic activity in terms of H3K27 methylation. The recurrent heterozygous somatic mutations of EZH2 include Y641F, Y641N, Y641H, and Y641S, which were identified in the germinal center B-cell-like (GCB) subtype of diffuse large B-cell lymphoma and follicular lymphoma^132,133^. Cells harboring both EZH2 Y641F/N and EZH2 A677G mutations exhibited significantly elevated levels of H3K27me3. Interestingly, this abnormal increase in H3K27me3 was not limited to lymphoma cell lines but was also demonstrated in primary lymphoma specimens. These findings provide additional evidence suggesting the impact of these mutations on the dysregulation of H3K27 methylation in lymphoma development and progression^131^.

It is crucial to distinguish the context in which EZH2 exerts antitumor or oncogenic effects in tumorigenesis. As shown in Fig. 3a, EZH2 acts as a tumor suppressor in LUAD, MDS, AML, T-ALL, and PMF. The mechanism underlying the tumor-suppressive effect of EZH2 in each of these cancer types is shown in Fig. 3d. Co-occurring EZH2 deletion and KRAS mutations exist in LUAD. Moreover, in blood cancers, inactivating mutations of EZH2 with TET2 have been observed in patients with myelodysplastic disorders, while in vivo deletion of EZH2 could increase the expression of “Myc module” genes and lead to myelodysplastic phenotypes.

The inactivation of EZH2 observed in patients is caused by EZH2 downregulation and mutation. Notably, EZH2 frameshift (fs) or deletion (del) mutations represent prevalent mutation types that prompt EZH2 downregulation and consequent aberrant protein function in patients with myelodysplastic syndrome (EZH2 K718fs) and T-ALL (EZH2 fs; K405fs, L674fs, D183fs, L728fs, K61fs, C606Y, and Q570fs, EZH2 del;(7)(q35q36.1) 142.67-150.97, 142.03-157.56, 143.92-156.84, and 143.33-158.82 Mb) (Fig. 3d). Point mutations within the D2, CXC, and SET domains of EZH2, including D664A and A255T in myelodysplastic syndrome and G266E, T393M, E549X, C606Y, and P577L in T-ALL, have been identified^118,134^. The enzymatic methyltransferase function of EZH2 primarily relies on its SET domain, although the N-terminal CXC domain adjacent to the SET domain also plays a crucial contributory role in facilitating this activity^135^. Mutations in the CXC and SET domains, such as D664A, E549X, C606Y, and P577L, disrupt the function of EZH2, contributing to the inhibition of its activity^136^. In addition, alterations in the D2 region, which overlaps with exon 8, have been associated with exon skipping and the production of out-of-frame EZH2 mRNA. This also underscores the potential impact of D2 mutations on mRNA expression and their significance in EZH2 loss-of-function^137^.

When EZH2 was inactivated in T-ALL, a proportional decrease in H3K27me3 levels was observed. Consequently, this decrease in H3K27me3 led to an increase in NOTCH1 signaling, which ultimately contributed to oncogenic transformation. The mutational sites associated with loss-of-function in patients with myelodysplastic syndrome or T-ALL do not overlap with the Y641 or A677 mutations previously identified as gain-of-function mutations. This finding suggests a clear distinction between the mutational profiles observed in these diseases.

### Other PKMTs exhibiting tumor-suppressive functions

Multiple studies have reported that several PKMTs, including mixed lineage leukemia (MLL)3, PRDM2/RIZ, PRDM5, SMYD4, SUV4-20H2, and EEF1AKMT3, exhibit tumor-suppressive functions rather than oncogenic roles. This section will discuss the tumor-suppressive properties of these PKMTs. In addition to the 8 representative PKMTs with tumor suppressor activity, PRDM16, MLL2, MLL4, SET domain bifurcated histone lysine methyltransferase 1 (SETDB1), SETD3, NSD1, NSD3, DOT1L, SUV39H1, and SUV39H2 have also been suggested to possess tumor suppressor activity^138–146^. However, their predominant function is to promote tumor growth, and the mechanisms underlying their potential tumor suppressor activity remain unclear, limiting their detailed discussion in this review.

#### MLL3

The MLL3 gene is located on chromosome 7q36 and encodes a protein with a length of 4911 amino acids. The gene includes several conserved domains and regions, such as DHHC, FYR, SET, post-SET domain, 7 coiled coil regions, WIN motif, and 10 zinc fingers (Fig. 2).

MLL3 has been identified as a haploinsufficient tumor suppressor mainly in certain subtypes of leukemia. The Cancer Genome Atlas dataset indicates that approximately 12% of AML patients exhibit a deletion in the MLL3 gene^147^. Moreover, research has shown that approximately 15% of FLT3-ITD-mutated AML patients exhibit recurrent MLL3 mutations or gene deletions, and patients with MLL3 mutations generally have a poor prognosis^148^. Consistently, MLL3 knockdown in FLT3-ITD-mutated cell lines or murine models promoted tumor growth^148^. However, low expression of MLL3 in cytogenetically normal AML does not appear to impact prognosis^149^. These findings suggest that MLL3 has tumor-suppressing functions that require cooperation with other events, such as TP53 inactivation. In fact, MLL3 deficiency in AML has been found to accelerate the development of myeloid leukemia under p53-deficient conditions and to impair the differentiation of hematopoietic stem and progenitor cells (HSPCs)^149^. Additionally, MLL3-downregulated leukemia was found to be resistant to conventional chemotherapy but sensitive to the bromodomain and extraterminal (BET) inhibitor JQ1^150^. Finally, somatic nonsense mutations in the MLL3 gene are accompanied by loss of heterozygosity (LOH) in human T-cell leukemia virus type I (HTLV-I)-associated acute adult T-cell leukemia (ATL). This finding highlights the critical role of MLL3 inactivation in the leukemogenesis of HTLV-1-induced ATL^151^.

The inactivation of MLL3 through frameshift mutations is common in CRC. Specifically, frameshift mutations in the poly A tract coding region of MLL3 are frequently identified in microsatellite-unstable CRC cells and primary tumors, causing loss of function of the MLL3 protein. According to these observations, it has been postulated that loss of MLL3 function may be an important contributor to the early development of CRC^152^.

Epigenetic changes due to the loss of MLL3 activity in bladder cancer cells result in the dysregulation of genes associated with the DDR and repair. Consequently, these cells impair the repair process of double-stranded DNA breaks through homologous recombination, leading to the accumulation of endogenous DNA damage and genomic instability. In addition, these cells substantially rely on PARP1/2 for DNA repair. Thus, it has been proposed that utilizing olaparib, a PARP1/2 inhibitor, as a therapeutic intervention can induce synthetic lethality in cells with low MLL3 expression^153^. Furthermore, inactivation of MLL3 methyltransferase activity in H3K4 in urothelial tumors has been reported to impede the p53-mediated DDR^154^.

#### PRDM2/RIZ

The PR domain zinc finger protein 2/RIZ (PRDM2/RIZ) gene is located on chromosome 1p36 and encodes a protein consisting of 1718 amino acids, which includes a PR/SET domain in the N-terminal region, an SH3-binding motif, and eight zinc finger motifs, which function as negative regulators of tumorigenesis (Fig. 2). Via an alternative promoter, the PRDM2/RIZ gene produces two distinct proteins, RIZ1 and RIZ2, with different motif configurations at the N-terminus. RIZ1, but not RIZ2, contains a PR domain^155^. Notably, RIZ1 knockout mice were shown to exhibit a high incidence of diffuse large B-cell lymphoma and the development of extensive tumors^156^. Additionally, RIZ1 is frequently expressed at low levels in various cancers, whereas RIZ2 is not. Thus, it is widely accepted that RIZ1 but not RIZ2 plays a crucial tumor-suppressive role^157^. Indeed, RIZ1 suppresses colony formation, proliferation, migration, and invasion and promotes apoptosis and cell cycle arrest at the G2/M phase through regulation of c-Myc, p53, AKT, and NF-κB in meningiomas, glioma, and breast cancer^158–162^.

The tumor-suppressive role of RIZ1 has been extensively studied in various types of brain cancers. RIZ1 expression is frequently downregulated in malignant meningioma tissues, while overexpression of RIZ1 suppresses cell proliferation and c-Myc expression, arrests the cell cycle in the G2/M phase, and induces apoptosis^158^. Furthermore, RIZ1 regulates the expression of UbcH10, a member of the ubiquitin-conjugating enzyme family known to possess oncogenic properties^163^, in a c-Myc-dependent manner^160^. Similarly, RIZ1 induces apoptosis and G2/M arrest in somatotroph adenoma cells through c-Myc regulation, and low RIZ1 levels in somatotroph adenoma patients are associated with poor prognosis in terms of tumor size, invasion, and recurrence^162^. In gliomas, particularly high-grade gliomas, RIZ1 expression is also reduced. RIZ1 expression shows a negative correlation with tumor grade, and high expression of RIZ1 leads to a favorable prognosis. Overexpression of RIZ1 consistently induces apoptosis and cell cycle arrest in the G2/M phase in human malignant glioma. Mechanistically, RIZ1 is thought to exert its tumor-suppressive effects in glioma by increasing p53 expression and suppressing NF-κB and AKT signaling^161^.

RIZ1 has been found to be genetically altered or downregulated in various types of cancer. For instance, genetic alterations of RIZ1, such as frameshift mutations, LOH, and promoter methylation, have been reported in 37% of primary gastric cancers^164^. Similarly, RIZ1 mRNA levels are often decreased due to hypermethylation of its promoter CpG island in breast (44%, 11/25) and liver (62%, 20/32) cancer specimens^165^. In addition, overexpression of RIZ1 in breast, liver, and colon cancer cells has been shown to induce G2/M cell cycle arrest and apoptosis^159,166,167^.

Nearly 33% (6/18) of endometrial cancer tissues harbor frameshift mutations in RIZ1^168^. Notably, RIZ1 expression is significantly reduced in estrogen receptor α (ERα)-positive endometrial cancer tissues, indicating a correlation between RIZ1 expression and ERα signaling. Indeed, estrogen reduced RIZ1 expression in an endometrial adenocarcinoma cell line, which facilitated tumor growth and tumor cell proliferation, migration, and invasion^169^.

The PRDM2/RIZ gene is situated on the 1p chromosomal region that is frequently deleted in testicular germ cell tumors (TGCTs)^170^. In addition, overexpression of RIZ1 promoted apoptosis and inhibited cell proliferation and colony formation in spermatogonial cells^171^, indicating a possible tumor suppressor role of RIZ1 in seminoma tumorigenesis. Similar to its effect in endometrial adenocarcinoma, estradiol leads to a decrease in RIZ1 transcript levels in spermatogonial cells. Additionally, RIZ1 binds to ERα in the presence of estradiol, which suggests that RIZ1 may regulate cell growth by mediating ERα signaling^171^. Finally, in CML, downregulation of PRDM2/RIZ expression is associated with promotion of cell proliferation, inhibition of apoptosis, and reduced differentiation, which are mediated by the dimethylation of H3K9 and subsequent suppression of the IGF-1 signaling pathway^172^.

#### PRDM5

The PRDM5 gene is located at chromosome 4q26 and encodes a protein comprising 630 amino acids that includes a PR/SET domain in its N-terminal region, followed by 16 zinc finger motifs^158^ (Fig. 2). In BRAF-mutated colorectal cancer, suppression of PRDM5 expression is associated with advanced stages across colorectal polyp subtypes of both the serrated and conventional pathways^173^. In gastric cancer, silencing of PRDM5 mediated by DNA methylation of the 5’ CpG island facilitated cancer cell growth^174^. Deng *et al*. demonstrated that PRDM5 induced G2/M-phase arrest and apoptosis in ovarian cancer, breast cancer, and hepatoma cell lines^175^. Loss of PRDM5 resulted in an increase in the numbers of adenomas and microinvasion foci by impairing gene expression in metabolic pathways in APCmin-driven intestinal adenomas^176^. Moreover, PRDM5, as a stress-responsive gene, was found to inhibit tumor cell growth and proliferation by aberrantly regulating the Wnt/β-catenin signaling pathway and oncogene expression in multiple tumors^177^.

#### SMYD4

SET and MYND domain-containing protein 4 (SMYD4) is composed of 804 amino acids, and the gene is located on chromosome 17p13. SMYD4 possesses a SET domain and a zinc finger motif (Fig. 2). The tumor suppressor function of SMYD4 has been documented in relation to breast cancer development^178^. SMYD4 suppresses tumorigenesis in breast cancer cell lines by mediating the downregulation of platelet-derived growth factor receptor α (PDGFR-α)^178^. This finding is consistent with the strong downregulation of SMYD4 in human breast cancer tissues, where its expression is positively correlated with patient relapse-free survival^179^. In addition, recent reports have shown that miR-1307-3p promotes the tumor response by inhibiting SMYD4 expression. High levels of miR-1307-3p are clinically linked to low SMYD4 expression, which leads to lower overall survival. Moreover, overexpression of miR-1307-3p in an in vivo model reduced SMYD4 expression and promoted the malignant transformation of mammary epithelial cells^180^. Furthermore, copy number alterations in the SMYD4 gene were observed in renal cell carcinoma^181^, and copy number alterations in SMYD4 in glioblastoma were associated with a poor prognosis^182^. However, the molecular mechanism regarding the tumor-suppressive effect of SMYD4 in those tumors is not fully understood.

#### SUV4-20H2

Suppressor of variegation 4-20 homolog 2 (SUV4-20H2) is a protein consisting of 462 amino acids located on chromosome 19q13 and containing a SET domain (Fig. 2). This protein functions as an H4K20 methyltransferase, predominantly contributing to the trimethylation of H4K20. In the context of cancer, SUV4-20H2 has been reported to act as an epigenetic regulator, promoting EMT in pancreatic cancer^61^. Despite its oncogenic role, SUV4-20H2 is widely recognized as a tumor suppressor.

The tumor-suppressive role of SUV4-20H2 has been extensively studied in breast cancer. Exogenous expression of SUV4-20H2 in breast cancer cells inhibited the metastatic potential of cancer cells by suppressing the expression of genes involved in cancer migration, such as tensin-3 and focal adhesion^183^. A recent study demonstrated that miR-29a, which is overexpressed in breast cancer stem cells, accelerates the migration, invasion, and EMT of breast cancer cells by targeting SUV4-20H2. Mechanistically, miR-29a suppresses the trimethylation of H4K20 via suppression of SUV4-20H2, which results in upregulation of connective tissue growth factor (CTGF) and early growth response protein-1 (EGR1)^184^.

Evidence supporting the tumor-suppressive role of SUV4-20H2 has been documented in various cancer types, including liver cancer, lung cancer, and osteosarcoma. In the context of hepatocarcinogenesis, SUV4-20H2 expression and H4K20 trimethylation levels are significantly reduced^185^. In lung cancer, loss of SUV4-20H2 and H4K20me3 causes cancer progression and indicates a poor prognosis, potentially by dysregulating telomere length maintenance^186^. Additionally, reduced expression of SUV4-20H2 has been observed in osteosarcoma cells, and RNA-seq analyses have suggested that the mitogen-activated protein kinase, p53, transforming growth factor, and ErbB pathways are downstream effectors of SUV4-20H2^187^.

#### EEF1AKMT3

EEF1AKMT3 is a member of the seven-beta-strand methyltransferase family and family 16 methyltransferases and is a lysine-specific methyltransferase. The EEF1AKMT3 gene is located on chromosome 12q14. EEF1AKMT3 has been identified as a tumor suppressor in gastric cancer. The expression of EEF1AKMT3 is significantly decreased in gastric cancer tissues vs. normal tissues in patients, and a low level of EEF1AKMT3 expression has been linked to a poor prognosis^188^. EEF1AKMT3 mediates the methylation of lysine residue 296 of the MAP2K7 protein, which has been implicated in tumor suppression. Mechanistically, the methylation of MAP2K7 by EEF1AKMT3 was shown to impede tumor activity by promoting the stabilization of TP53^188^.

### Potential therapeutic approaches targeting tumor-suppressive PKMTs

Several anticancer drugs that target protein lysine methyltransferases have been developed. Notably, chemical inhibitors targeting EZH2, G9a, and DOT1L have been developed. Tazemetostat (EPZ-6438, E7438) is an anticancer drug that targets EZH2 (Table 2). This drug acts as a competitive inhibitor, binding to the SAM-binding site within the SET domain of EZH2. Tazemetostat inhibits the methylation of the lysine 27 residue on histone H3, inducing apoptosis in lymphoma cells, thereby inhibiting its activity and suppressing the growth of non-Hodgkin lymphoma^189^.

**Table 2:** Inhibitors of EZH2 and their inhibitory potency.

Drug	Reported activity toward	IC_50_ value	Ref.
Tazemetostat (FDA-approved)	Metastatic or advanced epithelioid sarcoma (ES), elapsed or refractory (R/R) follicular lymphoma (FL) whose tumors are positive for an EZH2 mutation	EZH2 ➔ 11 nM	^204^
EL1	DLBCL	EZH2 ➔ 15 nM EZH2 Y641F ➔ 13 nM	^205^
GSK126	DLBCL	EZH2 ➔ 9.9 nM	^206^
CPI-360	Non-Hodgkin’s lymphoma (NHL)	EZH2➔ 0.5 nM EZH2 Y641N ➔ 2.5 nM	^207^
CPI-169	Non-Hodgkin’s lymphoma (NHL)	EZH2➔ 0.24 nM EZH2 Y641N ➔ 0.51 nM	^207^
EPZ005687	Lymphoma cells		^208^
EPZ011989	Mouse model of human B-cell lymphoma		^209^
ZLD10A	Non-Hodgkin’s lymphoma (NHL)	EZH2➔ 18.6 nM EZH2 Y641N ➔ 27.1 nM EZH2 A677G ➔ 0.9 nM	^210^
GSK503	DLBCL		^211^
JQEZ5	Human primary CD34+ chronic myeloid leukemia (CML) stem/progenitor cells	EZH2➔ 11.1 nM	^212^
GSK926		EZH2 ➔ 20 nM	^213^
GSK343	Breast cancer cells prostate cancer cells	EZH2 ➔ 4 nM	^213^
PF-06726304	Karpas-422 xenograft model		^214^
EZH2-IN-3	DLBCL	EZH2➔ 0.032 ± 0.019 nM	^215^
Lirametostat (CPI-1205)	Multiple myeloma and plasmacytoma cell models	EZH2➔ 2 nM	^216^
EBI-2511		EZH2 (A667G) ➔ 4 nM	^217^
UNC1999	DLBCL	EZH2 ➔ 2 nM	^218^
Valemetostat (DS-3201, DS-3201b	T-cell leukemia lymphoma cells (ATL cells)	EZH1/2 dual inhibitor (IC50 ≤ 10 nM)	^219^
(R)-ORS1	KARPAS-422 cells	EZH2 ➔10 nM	^220^
PF-06821497	Karpas-422 xenotransplantation		^221^

Most EZH2 inhibitors (Table 2) have been studied to demonstrate the suppression of EZH2 oncogenic functions. Notably, GSK126 (one of the EZH2 inhibitors) treatment in the KRAS-mutant NSCLC cell H358 and A549 resulted in an increase in phosphorylated AKT and ERK, potentially promoting cell proliferation^190–192^. However, treatment with GSK126 alone did not impact cell proliferation in these cells, leading the authors to suggest that EZH2 has dual roles at different stages of lung cancer depending on KRAS. Specifically, EZH2 suppressed the initiation of Kras-driven adenocarcinoma but promoted adenocarcinoma progression. Otherwise, it is also possible that each inhibitory chemical has another nonspecific activity affecting other responses to interfere with the tumor-suppressive function of EZH2. Given the pleiotropic role of EZH2 in cancer, it is imperative to develop a clear understanding of the mechanisms of EZH2 activators or inhibitors and to develop methods to increase the efficiency of these drugs.

A drug that targets G9a (histone-lysine N-methyltransferase 2) is A-366, which inhibits the activity of G9a, thereby impeding the proliferation and survival of cancer cells. A-366 inhibits the methylation of the lysine 9 residue of a histone, thereby inhibiting the growth and differentiation of leukemia^193,194^.

DOT1L, a histone methyltransferase, methylates lysine 79 in histone H3 and has emerged as a critical player in several tumors, including lung cancer, melanoma, neuroblastoma, liver cancer, and head and neck squamous cell carcinomas^195,196^. EPZ-5676, a potent and selective inhibitor of DOT1L, competes with the methyl-donating cofactor SAM and locks the SAM-binding pocket. Moreover, EPZ-5676 effectively disrupts histone methylation and suppresses the expression of MLL-fusion target genes^197^. Despite entering clinical trials based on this premise, this drug was found to have limited efficacy.

PKMT inhibitors hold immense potential as therapeutic agents for cancer treatment. While their direct application faces current limitations, innovative strategies such as combination therapies and alternative approaches targeting tumor suppressive PKMTs offer promising avenues to overcome these challenges^27^. Above all, alternative approaches, including miRNA-based regulation, focus on restoring the expression of tumor-suppressive PKMTs, including SETD2, to counteract tumorigenesis^67,198^. Enhancing the stability of methyltransferases is another strategy to maintain the activity of tumor-suppressive PKMTs and influence the epigenetic landscape and gene expression in cancer cells, thereby suppressing tumorigenic processes^199^. Extensive research and clinical investigations are necessary to fully explore the potential of PKMT inhibitors, thereby paving the way for personalized medicine and improved patient outcomes.

## Gene expression analysis of PKMTs in various cancers

**Fig. 4:**
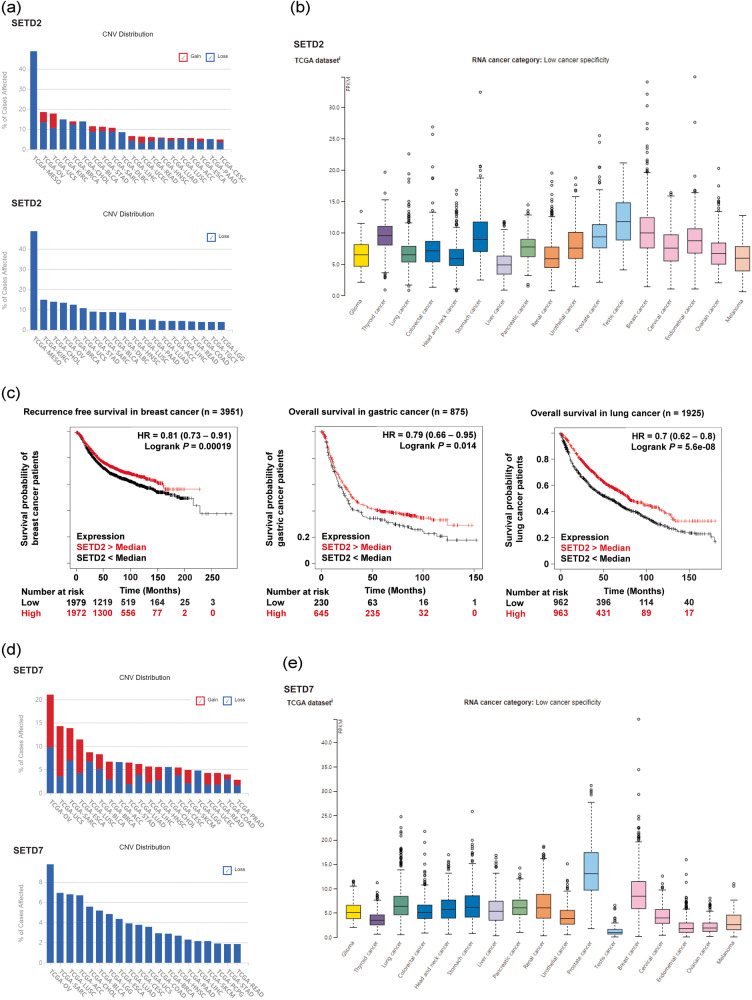

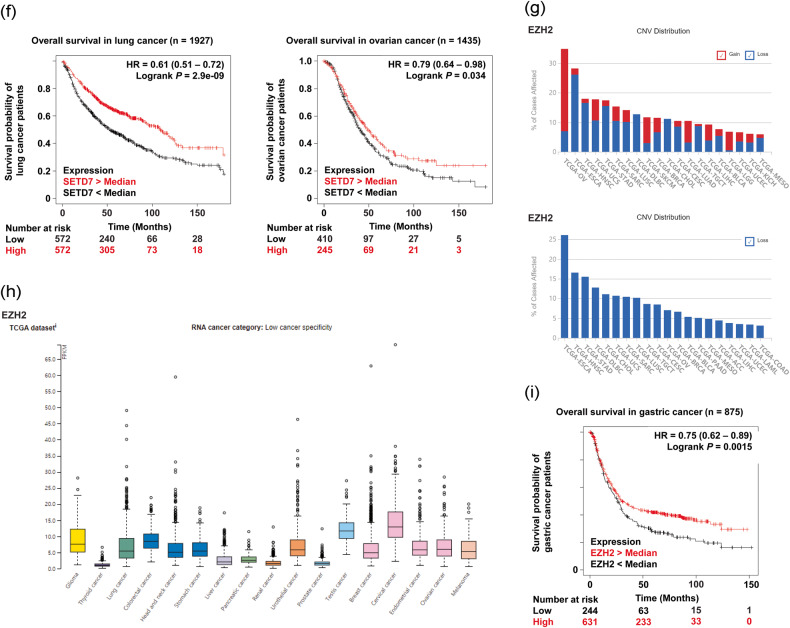

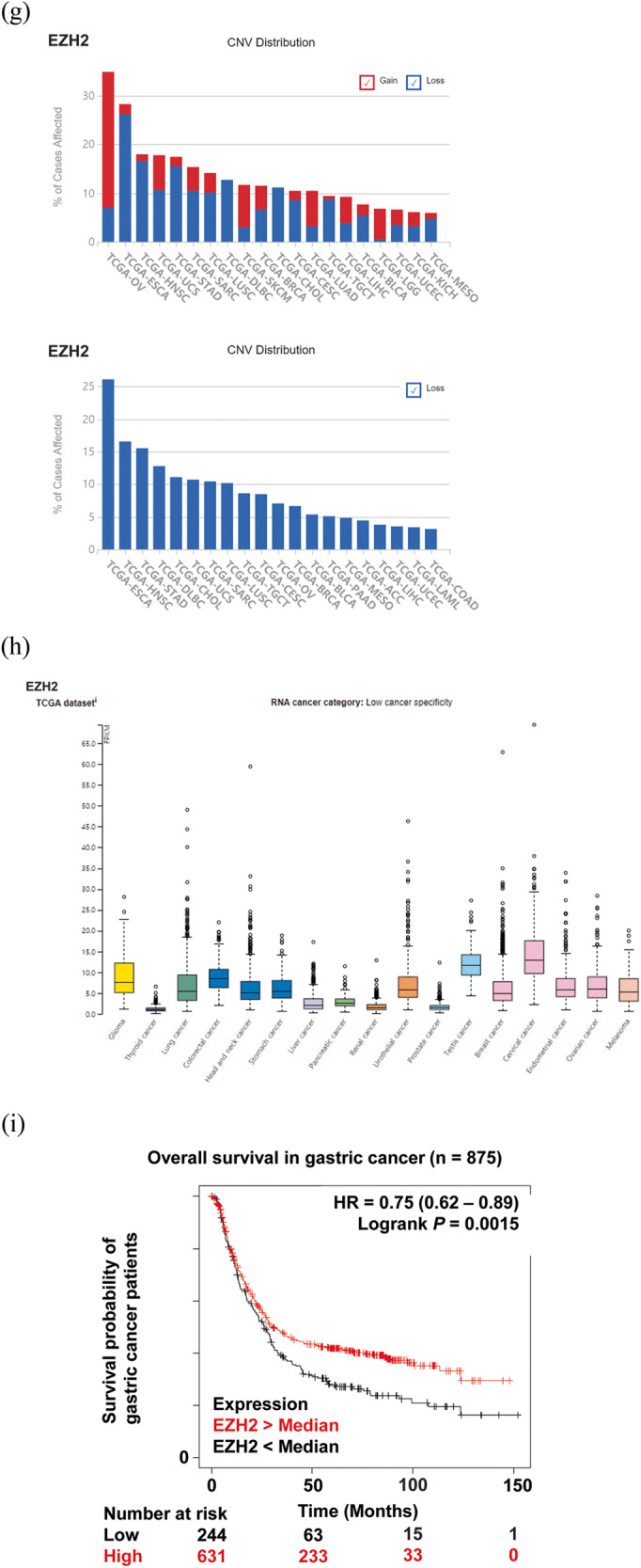
Gene expression analysis and clinical relevance of SETD2, SETD7, and EZH2. **a**, **d**, **g** A general pancancer overview of copy number variations (CNVs) in SETD2, SETD7, and EZH2, including copy number gain and copy number loss. The X-axis represents cancer type, while the Y-axis displays the frequencies of alterations (including both amplification and deletion) as percentages. The colors blue and red indicate deletion and amplification, respectively. **b**, **e**, **h** RNA expression profiles of SETD2, SETD7, and EZH2 based on RNA-seq data from The Cancer Genome Atlas. **c**, **f**, **i** Kaplan‒Meier analysis of recurrence-free survival (RFS) or overall survival (OS) based on the expression levels of SETD2, SETD7, and EZH2 in breast, gastric, lung, and ovarian cancers. ACC adrenocortical carcinoma, BLCA bladder urothelial carcinoma, BRCA breast invasive carcinoma, CESC cervical squamous cell carcinoma and endocervical adenocarcinoma, CHOL cholangiocarcinoma, COAD colon adenocarcinoma, DLBC lymphoid neoplasm diffuse large B-cell lymphoma, ESCA esophageal carcinoma, HNSC head and neck squamous cell carcinoma, KICH kidney chromophobe, KIRC kidney renal clear cell carcinoma, LAML acute myeloid leukemia, LGG lower-grade glioma, LIHC liver hepatocellular carcinoma, LUAD lung adenocarcinoma, LUSC lung squamous cell carcinoma, MESO mesothelioma, OV ovarian serous cystadenocarcinoma, PAAD pancreatic adenocarcinoma, PCPG pheochromocytoma and paraganglioma, PRAD prostate adenocarcinoma, READ rectal adenocarcinoma, SARC sarcoma, SKCM skin cutaneous melanoma, STAD stomach adenocarcinoma, TGCT testicular germ cell tumor, UCEC uterine corpus endometrial carcinoma, UCS uterine carcinosarcoma.

To gain a better understanding of tumor-suppressive PKMTs in various types of cancer and to provide valuable insights to aid in the development of novel therapeutic approaches targeting PKMTs, we conducted gene expression analyses of three representative PKMTs with established tumor-suppressive activity: SETD2, SETD7, and EZH2. We used several publicly available databases, including The Cancer Genome Atlas (TCGA) (https://www.cancer.gov.tcga), The Human Protein Atlas (https://www.proteinatlas.org), and Kaplan‒Meier Plotter (https://kmplot.com/analysis) (Fig. 4)^200–203^.

The majority of TCGA cancer types showed copy number loss of SETD2. Specifically, mesothelioma (48.81%, 41/84), kidney renal clear cell carcinoma (14.92%, 77/516), cholangiocarcinoma (13.89%, 5/36), ovarian cancer (13.5%, 79/585), breast cancer (12.5%, 134/1072), uterine carcinosarcoma (10.71%, 6/56), and gastric cancer (9.03%, 39/432), among others, showed downregulation (Fig. 4a). Although the observed downregulation of SETD2 in these cancer types suggests a potential role for SETD2 as a tumor suppressor, there is no existing research on the tumor suppressor activity or underlying mechanisms of SETD2 in cholangiocarcinoma, ovarian cancer, or uterine carcinosarcoma. Thus, we propose an exploration of the molecular mechanisms of SETD2 in these tumors. Similar to CNV analysis, TCGA RNA sequencing data indicated that the median values of fragments per kilobase of transcript per million mapped reads (FPKM) for SETD2 had low specificity in 17 cancer types (Fig. 4b). In particular, SETD2 mRNA downregulation was related to worse prognosis in terms of recurrence-free survival (RFS) in breast cancer (*p* < 0.001, *n* = 3951) and overall survival (OS) in gastric cancer (*p* = 0.014, *n* = 875) and lung cancer (*p* < 0.001, *n* = 1925) (Fig. 4c).

According to CNV distribution in the TCGA database, SETD7 shows copy number loss in ovarian cancer (9.74%, 57/585), sarcoma (6.92%, 18/260), lung squamous cell carcinoma (6.77%, 34/502), adrenocortical carcinoma (6.67%, 6/90), and other cancers (Fig. 4d). However, copy number gain of SETD7 was observed in ovarian cancer (11.28%, 66/585), uterine carcinoma (10.71%, 6/56), esophageal carcinoma (7.07%, 13/184), and sarcoma (6.92%, 18/260). In TCGA RNA sequencing data, the FPKM level of SETD7 showed low specificity in almost all cancer types except prostate and breast cancer (Fig. 4e). In addition, loss of SETD7 mRNA expression led to poor survival in ovarian cancer (*p* = 0.034, *n* = 1435) and lung cancer (*p* < 0.001, *n* = 1927) cohorts (Fig. 4f).

According to the TCGA database, EZH2 showed copy number loss in esophageal carcinoma (26.09%, 48/184), head and neck squamous cell carcinoma (16.51%, 86/512), gastric cancer (15.5%, 67/432), and lymphoid neoplasm diffuse large B-cell lymphoma (12.77%, 6/47) but a copy number gain in ovarian cancer (27.86%, 163/585), skin cutaneous melanoma (8.76%, 41/468), lung adenocarcinoma (7.41%, 38/513), and lower-grade glioma (6.24%, 31/497) (Fig. 4g). The mRNA expression level of EZH2 was decreased in thyroid, liver, pancreatic, renal, and prostate cancers compared with other cancer types in the TCGA database (Fig. 4h). Furthermore, EZH2-deficient gastric cancer patients had a shorter OS time than the controls (*p* = 0.0015, *n* = 875) (Fig. 4i).

Similar to previous research, our gene expression analysis also indicated that SETD7 and EZH2 exhibit both oncogenic and tumor-suppressive properties, depending on the cancer type. Thus, the design and development of anticancer drugs targeting these proteins require a meticulous approach and thorough investigations into their precise mechanisms in each cancer subtype.

## Conclusion

This literature review provides a summary of tumor-suppressive PKMTs, their substrates, and their functions in various human cancers at the molecular, cellular, and clinicopathological levels to help understand the tumor-suppressive ability of PKMTs and to provide new insights for the development of anticancer drugs targeting these proteins. There are approximately 18 tumor-suppressive PKMTs, and most of these PKMTs contain SET domains and share common features, including the ability to methylate histone proteins and subcellular localization in the nucleus. PKMTs predominantly exhibit tumor-suppressive activity by mediating the methylation of histones, α-tubulin, p53, and β-catenin. These modifications cause gene alterations in oncogenes and tumor suppressors. PKMTs may also contribute to genomic stability by regulating DNA repair processes or cell cycle arrest. Several PKMTs, including MLL3, PRDM2/RIZ, PRDM5, SMYD4, SUV4-20H2, and EEF1AKMT3, have been reported to exhibit tumor suppressor activities. However, few studies of these proteins have been performed, and their precise mechanisms and role in tumor suppression need to be further elucidated. The other group comprises PKMTs with both oncogenic and tumor-suppressive functions, including DOT1L, EZH2, MLL2, MLL4, NSD1, SETD2, SETD3, SETDB1, and PRDM16. The functions and molecular mechanisms of these proteins are relatively well documented, and they have been frequently reported as targets for cancer therapy. However, due to the pleiotropic roles of PKMTs in tumorigenesis, administering PKMT inhibitors can lead to unintended negative consequences. Therefore, it is important to carefully consider their use with regard to the cancer type and target enzyme.

## Data Availability

The datasets used and analyzed during the current study are available from the corresponding author upon reasonable request.
